# *Petunia* Floral Defensins with Unique Prodomains as Novel Candidates for Development of Fusarium Wilt Resistance in Transgenic Banana Plants

**DOI:** 10.1371/journal.pone.0039557

**Published:** 2012-06-22

**Authors:** Siddhesh B. Ghag, Upendra K. Singh Shekhawat, Thumballi R. Ganapathi

**Affiliations:** Plant Cell Culture Technology Section, Nuclear Agriculture & Biotechnology Division, Bhabha Atomic Research Centre, Mumbai, India; National Taiwan University, Taiwan

## Abstract

Antimicrobial peptides are a potent group of defense active molecules that have been utilized in developing resistance against a multitude of plant pathogens. Floral defensins constitute a group of cysteine-rich peptides showing potent growth inhibition of pathogenic filamentous fungi especially *Fusarium oxysporum in vitro*. Full length genes coding for two *Petunia* floral defensins, *PhDef1* and *PhDef2* having unique C- terminal 31 and 27 amino acid long predicted prodomains, were overexpressed in transgenic banana plants using embryogenic cells as explants for *Agrobacterium*–mediated genetic transformation. High level constitutive expression of these defensins in elite banana cv. *Rasthali* led to significant resistance against infection of *Fusarium oxysporum* f. sp. *cubense* as shown by *in vitro* and *ex vivo* bioassay studies. Transgenic banana lines expressing either of the two defensins were clearly less chlorotic and had significantly less infestation and discoloration in the vital corm region of the plant as compared to untransformed controls. Transgenic banana plants expressing high level of full-length *PhDef1* and *PhDef2* were phenotypically normal and no stunting was observed. In conclusion, our results suggest that high-level constitutive expression of floral defensins having distinctive prodomains is an efficient strategy for development of fungal resistance in economically important fruit crops like banana.

## Introduction

Banana (*Musa* spp.) is among the most important food crops in the world and constitutes the staple food for millions of people spread over different continents. India is the largest producer of bananas in the world with a total production of 31.89 million metric tones, which contributes approx. 34.5% of total production of top 20 banana growing nations [1]. As for any other crop plant, the yield and productivity of banana is constraint by the several biotic and abiotic stress determinants present in the immediate environment of the banana plant [2]. Among these, biotic stress is chiefly imparted by the numerous diseases and pests, which when present in an area above a limited threshold, preclude banana cultivation altogether. Chief among the banana diseases are fungal leaf diseases, vascular wilts, fruit rots and virus infestations like banana bract mosaic virus and banana bunchy top virus [3].

From a purely economical point of view, the most important among these is the Fusarium wilt (also called as the Panama disease). *Fusarium oxysporum,* which is responsible for causing wilt disease in numerous crop plants, is a soil borne ubiquitous species complex of plant pathogens that includes several *formae speciales*, each having a high degree of host specificity. *Formae speciales* are further sub-divided into races, based on pathogenicity towards a set of different cultivars within the same plant species [4]. Fusarium wilt in banana is caused by the infection and colonization of *Fusarium oxysporum* f. sp. *cubense* (Foc). Foc invades and occludes the xylem vessels of the roots leading to severe wilting of the banana plant [5]. The typical symptoms of the Fusarium wilt include progressive yellowing of the leaves, cracking of the pseudostem and discoloration of the corm tissue [6]. Yellowing proceeds from the oldest to the youngest leaves in the whorl. In due course, leaves of infected banana plants become bright yellow and finally start collapsing around the pseudostem [7]. Fungicides have not been found to be effective in controlling Foc. Foc chlamydospores can survive for decades in the soil and hence healthy banana plants can only be grown in soil which is free of the pathogen [6]. The disease was first reported in Australia in 1874 and it has now spread to all banana cultivating regions of the globe except Papua New Guinea, the South Pacific Islands and some countries situated around the Mediterranean [8].

Among the four recognized races of Foc, race 1, which was behind the epidemics in ‘Gros Michel’ plantations [5], also infects ‘Lady Finger’ (AAB) and ‘Silk’ (AAB) subgroups. Race 2 affects cooking varieties like ‘Bluggoe’ (ABB) and race 4 can attack race 1 and 2 susceptible as well as ‘Cavendish’ (AAA) varieties. Race 4 is thus the biggest concern as it threatens the major elite cultivars of banana. Recently, however a report has indicated that unstressed ‘Cavendish’ varieties can in fact, be attacked by race 1 Foc also [9]. This suggests that the pathogenicity towards elite ‘Cavendish’ cultivars could have evolved more than once. The above report also signifies the urgency which is required in developing cultivars resistant towards multiple races of Foc. As there are no natural sources of resistance against Foc known from any cultivated banana the only way forward for the development of Fusarium resistance in banana is through the incorporation of novel resistance genes derived from other organisms by using techniques of genetic engineering. Crop improvement by integration of suitable transgenes into elite, accepted cultivars overcomes many limitations of traditional breeding for disease resistance, including the possibility to use resistance genes from any organism and in special context of banana, the triploid nature and parthenocarpic fruit development of the most popular cultivars. Genes with antifungal functions (those coding for PR proteins, ribosome-inactivating proteins or small antimicrobial peptides like defensins) or which demonstrate antitoxin activities (like UDP-glucosyltransferase gene or alkaline lactonohydrolase) have been used to increase resistance to various pathogenic species belonging to genus *Fusarium*, including *F*. *culmorum*, *F*. *graminearum*, *F*. *verticillioides* and *F*. *oxysporum* [10].

**Figure 1:**
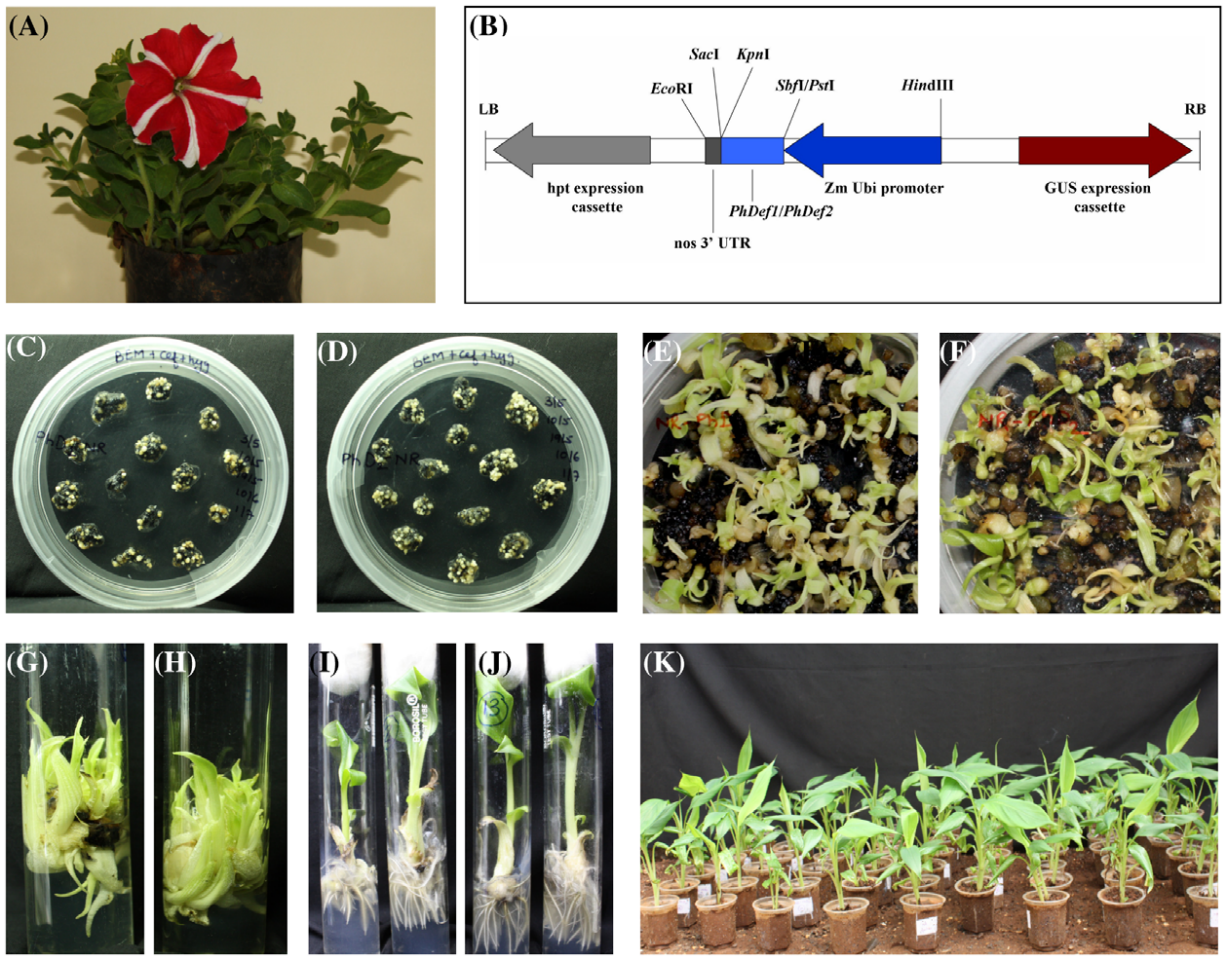
Genetic transformation of banana cv. *Rasthali* with full-length ***Petunia*** **defensin constructs.** (A) *Petunia* flower which served as the source of defensin genes. (B) T- DNA region of p*PhDef1*-1301/p*PhDef2*-1301 binary vector wherein *PhDef1* and *PhDef2* coding sequences were cloned downstream of *Zea mays* polyubiquitin promoter and upstream of nos (nopaline synthase) 3′ UTR in MCS of pCAMBIA-1301 vector. (C) and (D) Somatic embryos derived from p*PhDef1*-1301 and p*PhDef2*-1301 transformed cells on embryo induction medium 6 weeks post cocultivation. (E) and (F) Germination of transgenic somatic embryos derived from p*PhDef1*-1301 and p*PhDef2*-1301 transformed cells on medium supplemented with BAP 10 weeks post cocultivation. (G) and (H) Multiple shoots derived from p*PhDef1*-1301 and p*PhDef2*-1301 transformed cells on shoot induction medium 14 weeks post cocultivation. (I) and (J) Individual shoots derived from p*PhDef1*-1301 and p*PhDef2*-1301 transformed cells on root induction medium supplemented with NAA. (K) Hardening of rooted transgenic plants in the green house.

Incorporation of genes coding for anti-fungal peptides in transgenic banana plants has been attempted before. A synthetic substitution analogue of magainin, MSI-99 [11] and human lysozyme [12] were successfully expressed in transgenic banana plants which exhibited improved resistance toward both *Fusarium* and *Mycosphaerella* infection. Also, genes coding for an endochitinase, a stilbene synthase and a superoxide dismutase were stacked together in transgenic banana to obtain improved resistance against *Mycosphaerella* in limited filed trials [13]. A recent report has demonstrated that expression of apoptosis-inhibition-related animal genes (namely *Bcl*-*xL*, *Ced*-*9* and *Bcl*-*2* 3′ UTR) in transgenic banana plants leads to enhanced resistance towards Foc infection as indicated by significantly less internal and external disease symptoms [14].

Defensins constitute one of the largest families of small antimicrobial peptides in plants. Plant defensins are generally small basic proteins having 3–4 disulfide linkages. Defensins are particularly potent against fungal targets. Defensins interact with specific lipids on the fungal membrane and subsequently permeabilize them to inhibit fungus growth [15], [16], [17]. Defensins from radish seeds have been used to develop resistance against pathogenic fungi in many plant species. Recently, there has been great interest in studying defensins derived from floral tissues of plants. These defensins are characterized by the presence of C-terminal domains of up to 33 amino acids adjacent to the typical defensin domain of 47 or 48 amino acids. Three such floral defensins, NaD1, isolated from *Nicotiana alata* and PhD1 and PhD2, isolated from *Petunia hybrida* have been described in detail [18]. All these three floral defensins show potent antifungal activity against pathogenic strains of *Fusarium oxysporum*. Among them, PhD1 and PhD2 caused significant inhibition of *Fusarium oxysporum* f. sp. *dianthi* growth in vitro. PhD1 at 10 µg/ml showed 100% inhibition of hyphae growth whereas at the same concentration PhD2 gave 86% inhibition. Based on the relatively high potency of the *Petunia* floral defensins against pathogenic members of *F*. *oxysporum* complex and the presence of a anionic C-terminal prodomain responsible for neutralization of the cationic mature defensin domain and its vacuolar targeting, we hypothesized that the high-level expression of these defensins in vivo in transgenic banana plants will impart these plants effective resistance against Foc. The present study describes the expression of two *Petunia* floral defensins in transgenic banana plants followed by detailed bioassays for resistance against Foc infection.

## Materials and Methods

### Amplification of Defensin cDNAs

Total RNA was extracted from the freshly harvested *Petunia* flowers (Figure 1 A) using RNeasy Plant Mini kit (Qiagen, Germany). cDNA was synthesized using oligo (dT)_12–18_ primer and AccuScript Reverse Transcriptase (Invitrogen, USA). PCR amplification was carried out using Pfu Ultra AD DNA Polymerase (Stratagene, USA) using the following primers (from 5′ to 3′): *PhDef1* Fw: CCTGCAGGATGGCTCGCTCCATCTGTTTC, Rv: GGTACCCTACACCATCATATCTGCCTCAAGC; *PhDef2* Fw: CCTGCAGGATGGCTCGCTCCATCTGTTTC, Rv: GGTACCTTACTCCATCATATCTTCTTCGACCA. The following thermal cycling conditions were set for amplification: 94°C for 5 min followed by 30 cycles each with 94°C for 1 min, 55°C for 1 min and 72°C for 1 min with a final extension 72°C for 10 min. The amplified products were gel purified and subsequently sequenced.

**Figure 2:**
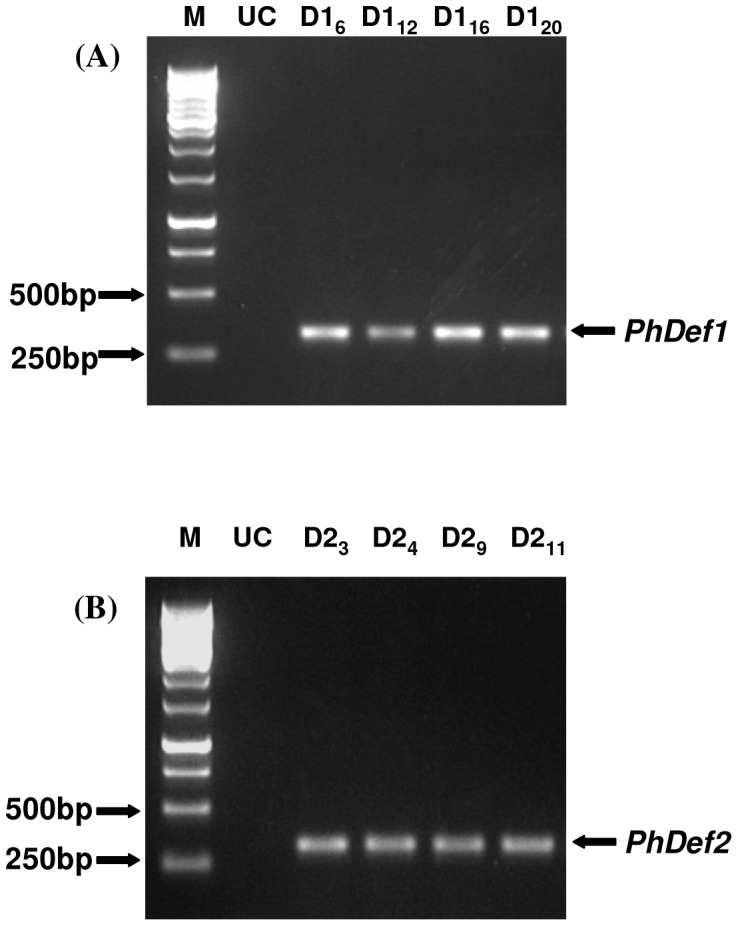
Genomic DNA PCR analysis of transgenic plants. Genomic DNA isolated from selected transgenic lines derived from the two constructs and untransformed control (UC) plants was used in PCR analysis using primers specific to *PhDef1* and *PhDef2*. (A) p*PhDef1*-1301 derived transgenic lines. (B) p*PhDef2*-1301 derived transgenic lines.

### Plasmid Construction and Agrobacterium Transformation

The two amplified products *PhDef1* and *PhDef2* were cloned in pCAMBIA-1301 binary vector (Genbank accession no. AF234297) as part of a constitutive expression cassette having *Zea mays* polyubiquitin promoter and nos 3′ UTR by three way ligation reaction (performed as essentially described in [19]) to yield binary vectors p*PhDef1*-1301 and p*PhDef2*-1301. The newly constructed binary vectors were subsequently mobilized into *Agrobacterium tumefaciens* strain EHA 105 by electroporation before being used to transform embryogenic cells of banana.

### Plant Transformation, Selection and Regeneration of Transformed Tissues

A single *Agrobacterium* colony was inoculated in liquid YENB medium (0.75% w/v yeast extract and 0.8% w/v nutrient broth) supplemented with 50 mg/l kanamycin and incubated overnight at 27°C with an orbital shaking of 180 rpm. *Agrobacteria* from the overnight grown culture were resuspended in the same medium with kanamycin at an OD_600 nm_ of ∼0.1 and grown for another 4–5 hours under the same conditions until an OD_600 nm_ of ∼0.6–0.8 was attained. The suspension was centrifuged at 6,500 *g* for 15 min and resuspended in M2 medium [MS salts, 2,4-D (1 mg/l), biotin (1 mg/l), malt extract (100 mg/l), glutamine (100 mg/l) and 4.5% sucrose] [20] added with 100 µM Acetosyringone (ACS) to a final density of OD_600 nm_ ∼0.2. The bacterial suspension so obtained was used for cocultivation with banana embryogenic cell suspension cultures as described earlier [21]. Embryogenic cells of banana (0.5 ml PCV) were sieved through an 85 µm sieve and cocultivated with *Agrobacterium* for 30 minutes. After cocultivation the cells were aspirated onto glass filter discs and were transferred onto semi-solid M2 medium supplemented with 100 µM ACS. The plates were incubated in dark for three days at 23±2°C. Then, the filters along with the cells were transferred to fresh semi-solid M2 medium supplemented with cefotaxime (400 mg/l). Three days later the cells were transferred to banana embryo induction medium added with cefotaxime (400 mg/l) and hygromycin (5 mg/l). Developed embryos were transferred onto selection medium and subcultured for three rounds after every three weeks. Matured embryos were transferred to MS medium supplemented with lower concentration of BAP (0.5 mg/l) for shoot emergence. The germinating embryos were transferred to banana multiplication medium for multiple shoot induction [22]. Single shoots were isolated and rooted on MS medium supplemented with NAA (1 mg/l). The rooted plantlets were hardened in the greenhouse and used for further analysis.

**Figure 3:**
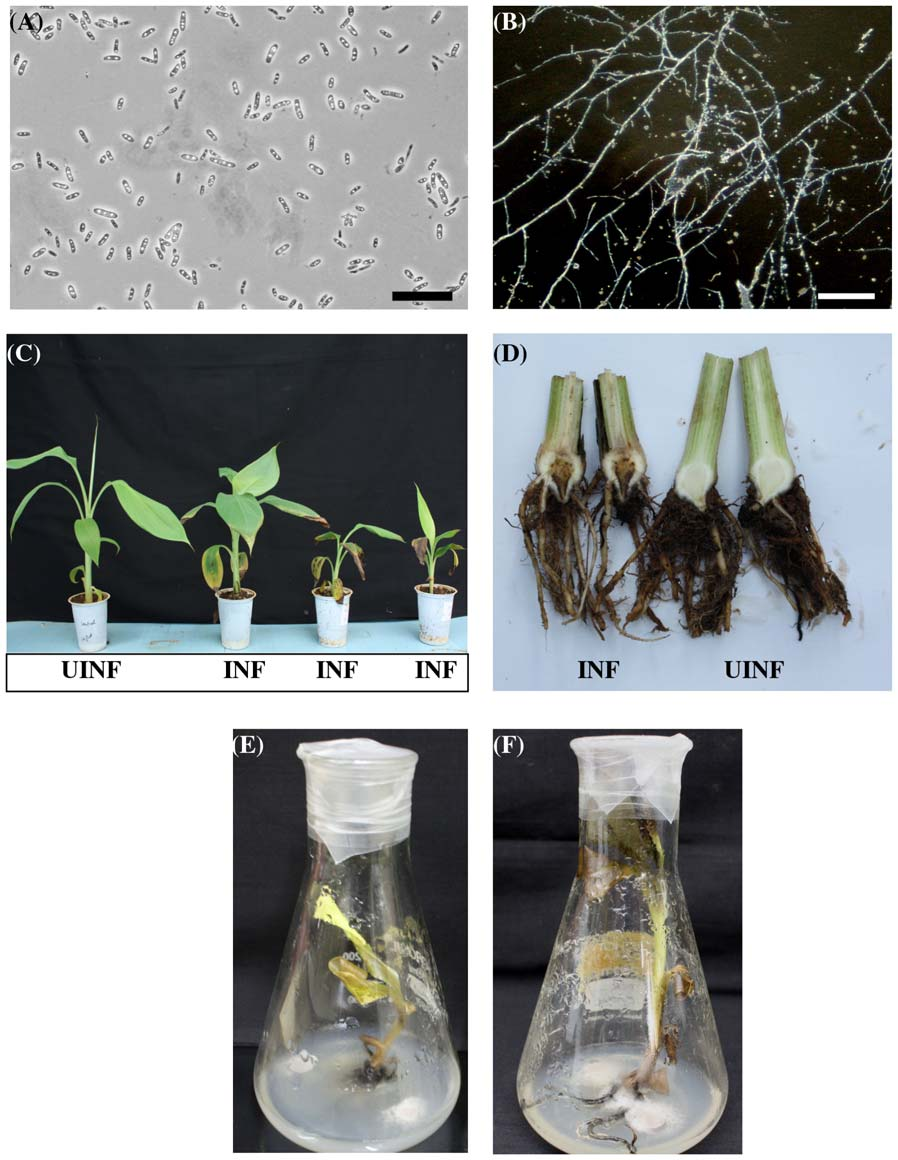
Identification and pathogenicity testing of the Foc isolate. Foc oval shaped spores (A) and mycelium (B) as observed under microscope. Bars correspond to 25 µm (C) Hardened plants of banana cv. *Rasthali* showing initial symptoms of Foc race 1 infection 3 weeks post inoculation (INF). An uninfected control banana plant is shown on the extreme left (UINF). (D) Necrotic lesions developed inside the corm subsequent to Foc race 1 inoculation on banana cv. *Rasthali* (left). An uninfected control banana plant is shown on the right. Foc infected untransformed control plants showing disease symptoms after 2 weeks (E) and 4 weeks (F). Mycelium is seen covering the roots of the infected plant.

### Molecular Analysis of the Transformants - Genomic DNA PCR

Putative transformants selected based on the preliminary GUS staining (performed as described in [23]) and their growth on hygromycin containing medium were analyzed using PCR to check the presence of the T-DNA. Genomic DNA was isolated from young banana leaves using GenElute Plant Genomic DNA Miniprep Kit (Sigma, USA) and was used as template in PCR reactions using primers specific for *PhDef1* and *PhDef2* coding sequences. The PCR cycling conditions used were the same as mentioned before. Genomic DNA isolated from untransformed banana plant was used as a control in these PCR reactions.

### Characterization of Foc Accession by ITS Sequencing

Foc isolate was obtained from Indian Institute of Horticulture Research (IIHR), Bangalore (India). The isolate was grown on solid Potato Dextrose Agar medium for 7 days at 25°C. Genomic DNA was isolated as described by [24] and used for PCR. Characterization of the isolate was done at the molecular level by sequencing the ITS (inter transcribed spacer) region sequence. Partial ITS sequence was amplified using primers ITS 1 and ITS 4 [25] and subsequently sequenced. Blast analysis tool was used to identify the isolate by homology matching. Race1 susceptible banana cultivars were infected with *Fusarium* spores to check for its race specific pathogenicity.

### Evaluation of Transgenic Banana Plants for Resistance against Foc - *in vitro* Bioassay

Foc was cultured on potato dextrose broth for 5 days at 30°C for obtaining spores. Rooted banana plants having minimum five fresh leaves and 3 white roots were selected for infection [26]. Plants were transferred to 250 ml Erlenmeyer flasks with semi-solid medium containing half strength MS salts and 0.8% agar. Two paper discs of 7 mm diameter were dipped in spore suspension (10^6^ spores/ml) and placed on the medium. Flasks were sealed with parafilm and incubated under normal culture conditions. The plants were observed after 4 weeks for Foc infection symptoms such as yellowing of leaves, pseudostem discoloration/cracking and wilting of leaves. A four point disease rating scale was used to ascertain the relative severity of Foc symptoms: no symptoms (no indication of any physiological distress); Mild (marginal yellowing and wilting of smaller leaves at the base); Moderate (more than half of the pseudostem discolored and half of the leaves wilted) and Severe (whole plantlet wilted). For untransformed controls and each of the selected transgenic line a minimum of four replicates were screened for Foc infection and representative plants were photographed.

### Evaluation of Transgenic Banana Plants for Resistance against Foc - *ex-vivo* Bioassay

Foc cultures were grown in Potato dextrose broth for 5 days at 30°C followed by separation of spores using four layers of cheesecloth. Spore concentration was adjusted to a spore count of 8×10^5/^ml using haemocytometry. For plant bioassay, fungal spores (500 ml) were inoculated in an autoclaved mixture (10 kg) of sand and maize bran in ratio of 19:1. The mass culture was incubated at room temperature for 4 weeks. Two months old hardened plants were used for infection by replanting them in a mixture of soil and mass culture (1∶1) [11]. Untransformed banana plants were used as control for these experiments. A four point disease rating scale used as above was utilised to determine the relative severity of Foc symptoms 6 weeks post infection. For untransformed controls and each transgenic line a minimum of four replicates were screened for Foc infection and representative plants were photographed intact and after longitudinal cutting.

**Figure 4:**
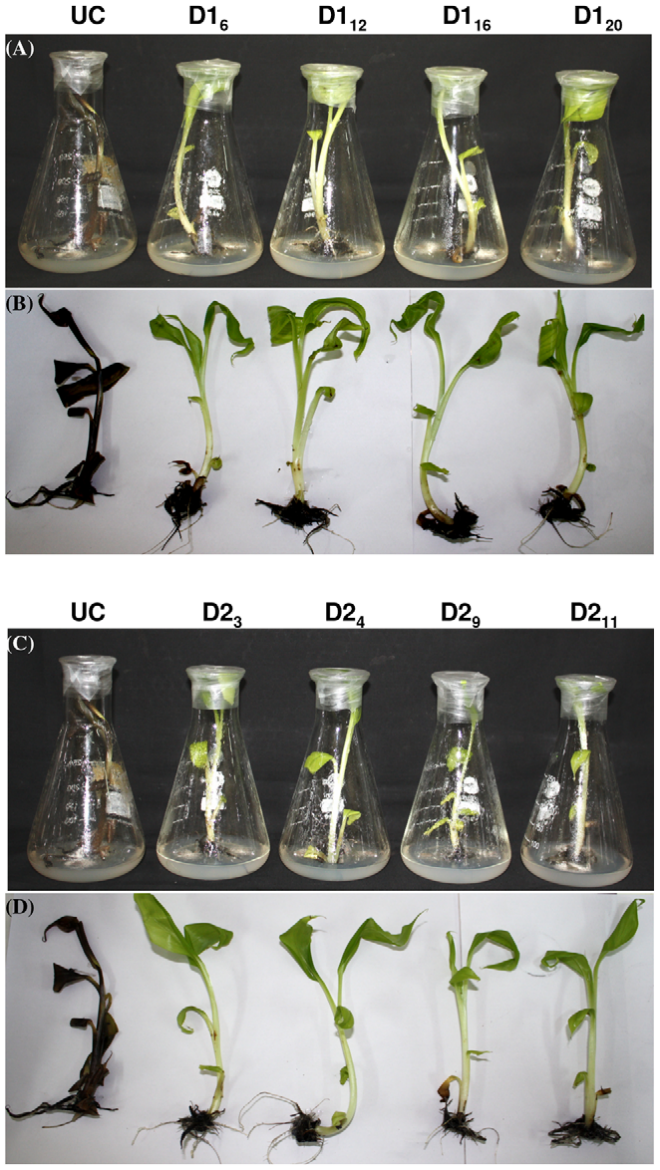
In vitro bioassay for Foc resistance in transgenic banana plants. In vitro bioassay was performed wherein p*PhDef1*-1301 (A and B) and p*PhDef2*-1301 (C and D) transgenic plants and untransformed control banana plants (UC) were challenged using Foc spore suspension. Four weeks post inoculation, untransformed control plants died whereas transgenic plants survived and showed normal growth.

### Evaluation of Transgenic Banana Plants for Resistance against Foc - Microscopic Examination

Corm sections of transgenic banana plants were examined using light microscope (Eclipse 80i, Nikon, Japan) following infestation by Foc. Uninfected and infected banana plants were used as control for microscopic studies.

### Molecular Analysis of the Transformants - Southern Blot Analysis

Confirmation of transgenic nature and T-DNA copy number determination was performed by southern blot analysis. Genomic DNA (∼20 µg) was digested with restriction enzyme *Kpn*I overnight at 37°C. The digested DNA fragments were then purified using High Pure PCR Product Purification Kit (Roche Applied Science, Germany) after deactivation of the restriction enzyme at 80°C for 20 minutes. Further the purified DNA was separated first at field strength of 3–4 V/cm for 1 hour and then overnight at 1.25 V/cm in a 0.9% (w/v) agarose TAE gel. DNA was transferred onto positively charged nylon membrane and subsequently immobilized by baking at 120°C for 30 min. DIG-labeled probes (Roche Applied Science, Germany) were generated using hygromycin coding sequence. Prehybridization and hybridization steps were carried out at 45°C for two hours and overnight respectively. The nylon filter was then washed twice for 15 minutes each with 2 X SSC, 0.1% (w/v) SDS at room temperature followed by two washes each with 0.5 X SSC, 0.1% (w/v) SDS at 65°C. Chemiluminescent detection of hybridization signals was performed using DIG High Prime DNA Labeling and Detection Starter Kit II (Roche Applied Science, Germany).

### Molecular Analysis of the Transformants - Genomic Integrity of p*PhDef1* and p*PhDef2* Expression Cassettes in Banana Plants

To investigate the intactness of p*PhDef1* and p*PhDef2* expression cassettes in transgenic banana plants, genomic DNA from transgenic lines and untransformed control plants were digested using *Eco*RI and separately with *Hin*dIII and *Kpn*I enzymes. DIG-labeled probes generated using *PhDef1* and *PhDef2* coding sequences were used to probe the digested DNAs to demonstrate the genomic integrity of the transferred T-DNAs. Genomic DNA digestion, transfer to nylon filter and detection of hybridization signals was performed as described above with DIG High Prime DNA Labeling and Detection Starter Kit II (Roche Applied Science, Germany).

### Molecular Analysis of the Transformants - RT-PCR Analysis

Total RNA was extracted from young transformed banana leaves and untransformed control leaves using Concert Plant RNA Reagent (Invitrogen, USA). It was then cleaned up using RNeasy Plant Mini Kit (Qiagen, Germany) with on-column DNase digestion. This RNA (∼5 µg) was then used to make first strand cDNA using Oligo (dT)_12–18_ primer (Invitrogen, USA) and ThermoScript Reverse Transcriptase (Stratagene, USA) according to manufacturer’s instructions. Primers specific for *PhDef1* and *PhDef2* coding sequences were used in PCR reactions performed under the same thermal cycling conditions as before. The PCR products were then analyzed on 1% agarose gel.

### Molecular Analysis of the Transformants - Northern Blot Analysis

Total RNA was extracted from banana leaf tissue as described above and separated in a 1.2% FA-MOPS agarose gel. The samples were allowed to resolve in 1X MOPS buffer at field strength of 3–4 V/cm for 2 hours. Further it was soaked twice each for 15 min with 20X SSC (to ensure efficient equilibration) and then transferred onto positively charged nylon membrane. The membrane was baked at 120°C for 30 min. DIG-labeled DNA probes generated using *PhDef1* and *PhDef2* coding sequences were allowed to probe the membrane. Prehybridization was carried out at 50°C for 2 hours followed by hybridization with DIG-labeled probes at 50°C overnight. Membrane was washed twice each with 2X SSC; 0.1% (w/v) SDS at room temperature and 0.5X SSC; 0.1% (w/v) SDS at 60°C. Chemiluminescent detection of hybridization signals was performed as indicated in Southern blot analysis.

**Figure 5:**
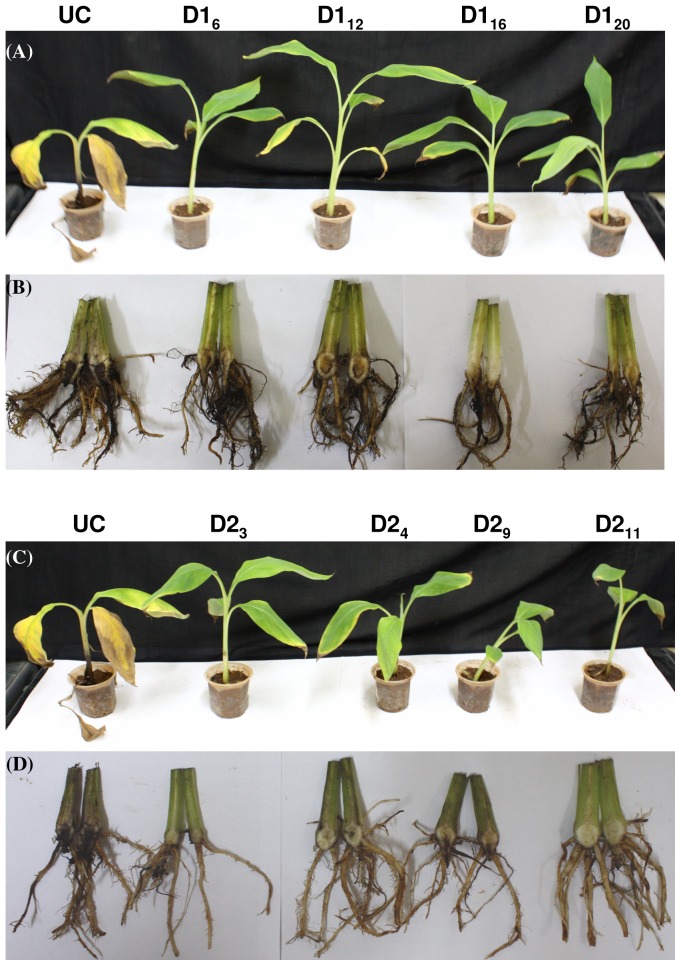
Ex vivo bioassay for Foc resistance in transgenic banana plants. p*PhDef1*-1301 and p*PhDef2*-1301 transgenic plants and untransformed control banana plants hardened in a greenhouse for 2 months were challenged with Foc mass culture. External and internal symptoms of Foc infection on p*PhDef1*-1301 (A and B) and p*PhDef2*-1301 (C and D) transgenic and untransformed control banana plants (UC). Untransformed control plants showed yellowing of leaves and wilting whereas transgenic plants showed negligible infection. Corm sections of control plants showed intense discoloration whereas the transgenic plants showed significantly less discoloration of the corm tissue.

**Figure 6:**
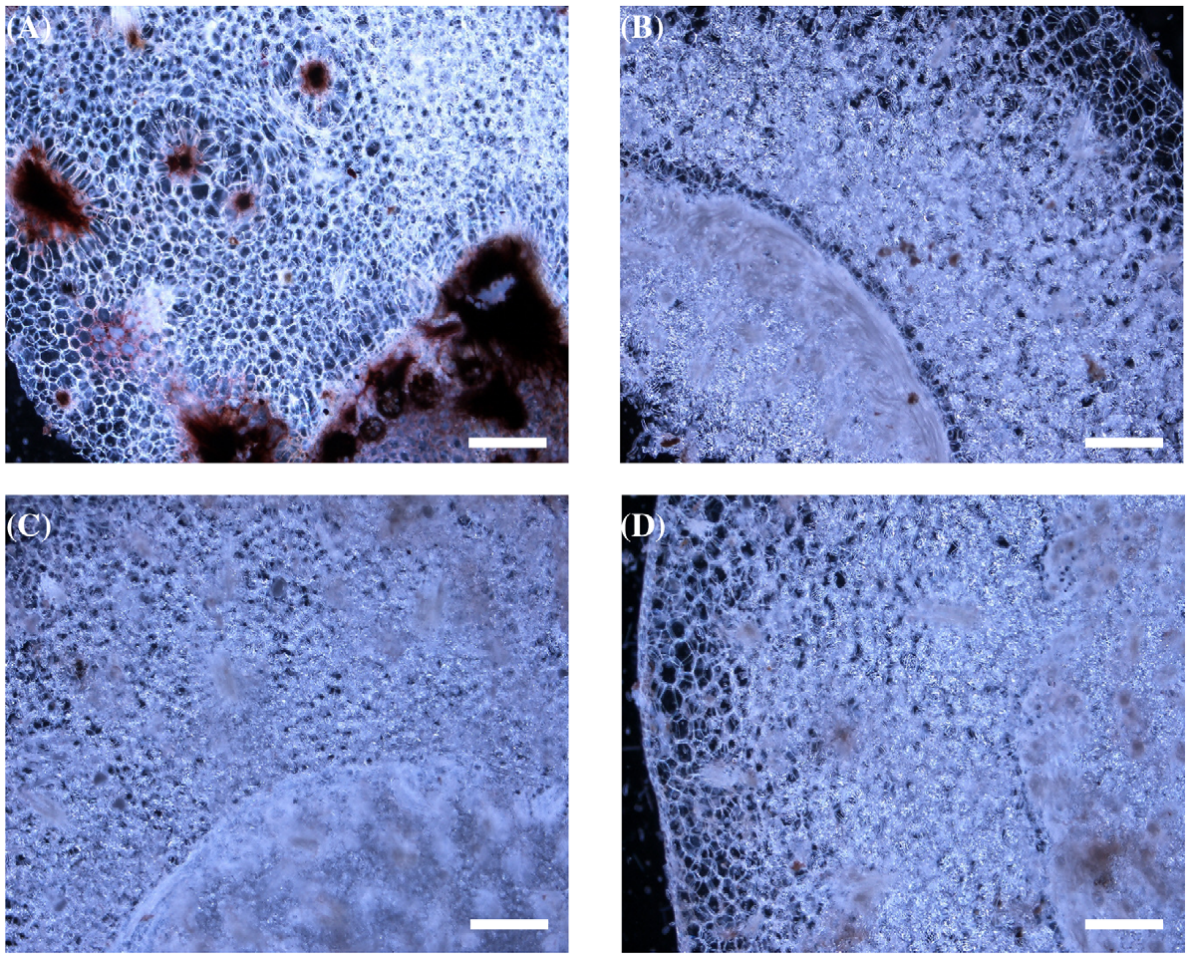
Microscopic studies of the Foc infection in control and transgenic banana plants. Untransformed control banana plants infected with Foc showed intense discoloration of corm vascular tissue (A) as compared to corm of uninfected control plants (B). Transgenic plants expressing *PhDef1* (C) and *PhDef2* (D) show negligible discoloration of the tissue. Bars correspond to 250 µm.

**Figure 7:**
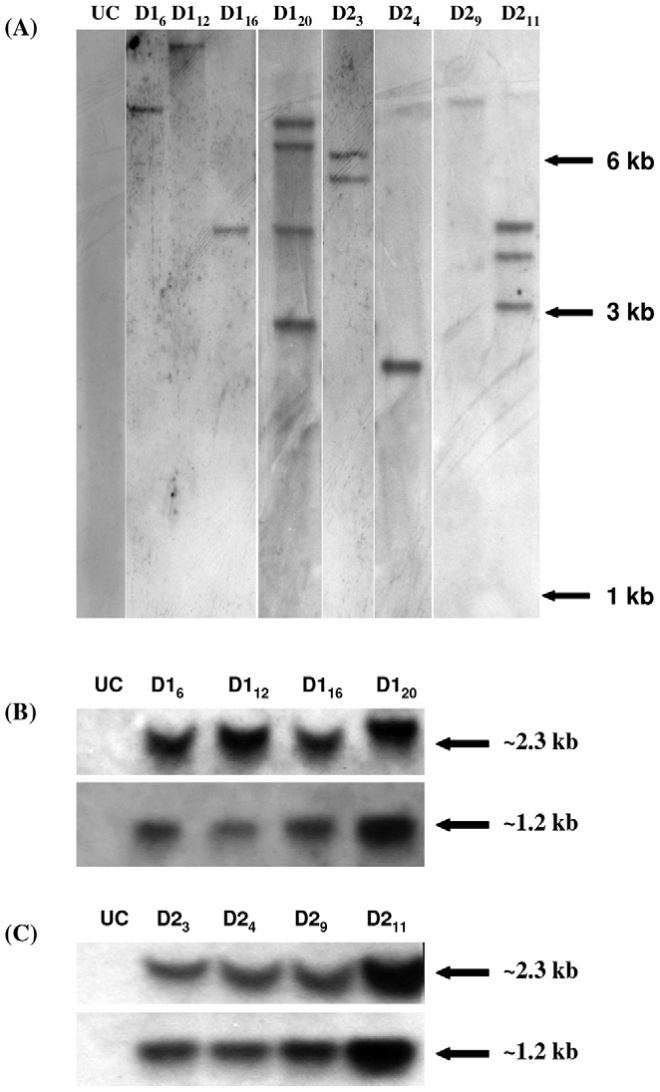
Determination of Copy number and genomic integrity of T-DNA in transgenic banana plants. (A) Southern analysis to determine the transgene copy number in transgenic banana plants expressing *PhDef1* (D1_6_, D1_12_, D1_16_, D1_20_) and *PhDef2* (D2_3_, D2_4_, D2_9_, D2_11_). UC denotes an untransformed control plant. Genomic DNA digested with *Kpn*I was allowed to hybridize with DIG-labeled probes targeted against hygromycin phosphotransferase gene present in the T-DNA of the two binary vectors used for genetic transformation. Genomic DNA digested with *Eco*RI and *Hin*dIII + *Kpn*I and probed with p*PhDef1* (B) and p*PhDef2* (C) coding sequences.

**Figure 8:**
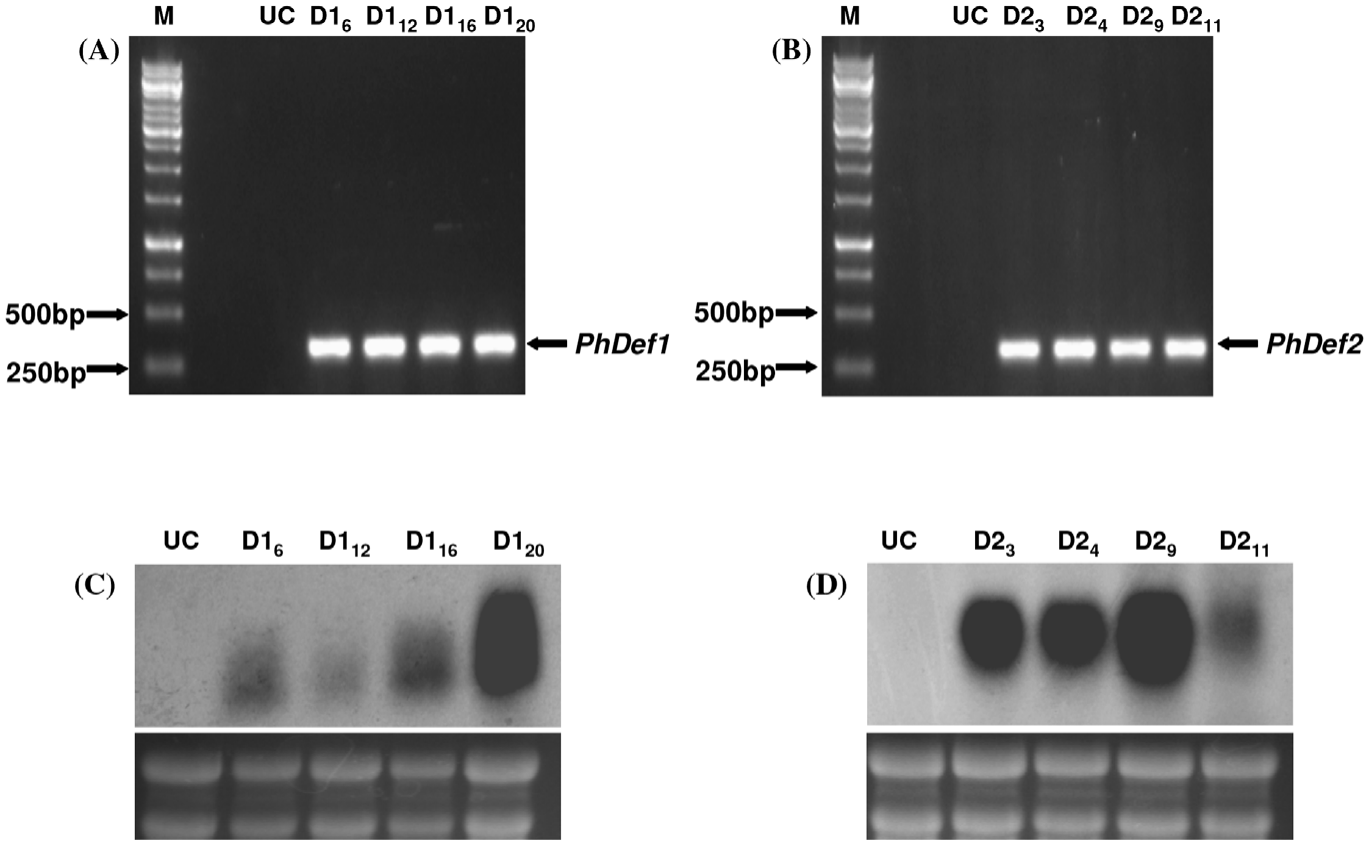
Expression analyses of the transgenic banana plants. (A) and (B) RT-PCR of selected p*PhDef1*-1301 and p*PhDef2*-1301transgenic lines performed using primers specific to *PhDef1* and *PhDef2*. (C) and (D) Northern blot analysis of selected p*PhDef1*-1301 and p*PhDef2*-1301 transgenic lines performed using FA-MOPS gel. Total RNA isolated from the transgenic leaves was filter hybridized with DIG labeled DNA probes targeted against *PhDef1* (C) and *PhDef2* (D) gene. Note the high-level expression of *Petunia* defensins in the different transgenic lines. rRNA bands stained using ethidium bromide indicate equal loading of total RNA in different lanes.

## Results

### Isolation, Cloning and Generation of Transgenic Lines

cDNAs for *Petunia hybrida* defensins namely *PhDef1* (312 bp) (Genbank Accession No: HQ694498) and *PhDef2* (306 bp) (Genbank Accession No: HQ694499) were successfully amplified using fresh flower tissues, cloned into a constitutive expression cassette in the MCS of pCAMBIA 1301 binary vector and then sequenced (Figure 1 B). Three distinct domains namely the N-terminal signal sequence (25 amino acids in both PhDef1 and PhDef2), the active defensin domain and a C-terminal prodomain predicted to play a part in intracellular trafficking are evident from the predicted peptides sequences of the two defensins. PhDef1 consists of 47 amino acids long defensin domain and 31 amino acids long C-terminal prodomain whereas PhDef2 has a 49 amino acids long defensin domain and a 27 amino acids long C-terminal prodomain.

Banana embryogenic suspension culture cells were separately transformed using *Agrobacterium tumefaciens* EHA 105 strain harboring p*PhDef1*-1301 and p*PhDef2*-1301. The transformed cells were grown on M2 medium supplemented with ACS in dark for 3 days and further cultivated on banana embryo induction medium added with cefotaxime and hygromycin. Transformed cells were GUS stained five days post cocultivation. Characteristic intense blue coloration in majority of the cells pointed out to good transformation efficiency. Whitish embryos developed on the medium (Figure 1 C, D) were subcultured for three subsequent rounds on hygromycin supplemented medium. Embryos germinated on a medium with low concentration of BAP (Figure 1 E, F). Germinated embryos were then transferred onto shoot multiplication medium (Figure 1 G, H) and then rooted in MS medium supplemented with NAA (Figure 1 I, J). Eventually, the plantlets obtained were hardened in greenhouse (Figure 1 K).

Out of a total of 24 and 28 GUS positive, hygromycin tolerating transformed banana lines obtained for p*PhDef1*-1301 and p*PhDef2*-1301 constructs respectively, four transgenic lines each were selected based on the intensity of GUS staining and growth vigor in hygromycin supplemented medium. PCR analysis of these four selected *PhDef1* and *PhDef2* overexpressing transgenic lines, performed using specific primers, resulted in the amplification of single DNA products corresponding to *PhDef1* and *PhDef2* coding sequences (Figure 2 A, B). These fragments were found to be absent in untransformed control plants.

### Characterization of Foc Accession by ITS Sequencing

*Fusarium* isolate was positively identified as Foc through ITS1–5.8S–ITS2 region sequencing. Using ITS1 and ITS4 primers we could amplify a 544 bp product which was subsequently sequenced (Genbank Accession No: HQ694500). This isolate when cultured on PDA for 7 days at 25°C showed extensive pink colored mycelium growth. Mycelial growth and spores were observed under light microscope (Figure 3 A, B). Further, pathogenicity studies were carried out wherein AAB group banana (cv. *Rasthali*) plants were infected by *Fusarium* spore suspension and incubated under green house conditions. After 4 weeks, plants started showing distinctive wilt symptoms such as yellowing of the older leaves, pseudostem cracking and discoloration of the corm tissue (Figure 3 C, D). After six weeks complete wilting of the plant was observed. The present isolate could successfully infect AAB group banana (cv. *Rasthali*) plants, but was unable to infect AAA group banana (cv. *Grand Nain*) plants, indicating that it probably belongs to Race1 of Foc (Data not shown). In vitro infected untransformed control plants (cv. *Rasthali*) showed typical disease symptoms such as discoloration of the pseudostem and wilting of the leaves after 2 weeks (Figure 3 E). Eventually complete wilting was observed after 4 weeks of Foc inoculation (Figure 3 F).

### Enhanced Resistance of Transgenic Banana Plants

To study the response of the four selected transgenic banana lines expressing *Petunia* floral defensins towards Foc challenge, the transgenic lines and untransformed control plants were inoculated with a confirmed pathogenic strain of Foc characterized by ITS sequencing in both in vitro and ex vivo bioassay conditions. For in vitro bioassay (Figure 4), both transgenic groups along with untransformed control plants were injured in the root region and transferred to half strength MS medium added with 0.8% (w/v) agar. Paper discs holding Foc spore suspension (10^6^ spores/ml) were allowed to grow in the medium. Foc spores germinated and the mycelium growth was seen covering the roots. Two weeks later control plants clearly showed the disease symptoms and got wilted four weeks post inoculation, whereas the selected transgenic lines of p*PhDef1*-1301 and p*PhDef2*-1301 did not showed any symptoms. These transgenic lines showed neither yellowing of the leaves nor browning of the pseudostem.

To determine the performance of transgenic banana plants under Foc challenge in green house conditions, four lines of each of the two defensin constructs and untransformed control plants were transplanted in 1∶1 mixture of soil and fungal mass culture. Plants were monitored for visible symptoms every week. After 4 weeks control plants showed typical yellowing of the older leaves and cracking of the pseudostem whereas transgenic plants showed no symptoms (Figure 5). Yellowing of the leaves began from the margin of the leaf lamina progressing towards the midrib region, which was followed by further browning and falling off from the pseudostem. Six weeks later, there was complete wilting of untransformed control plants whereas the transgenic plants had only mild symptoms like yellowing at the margin of the leaves and partial splitting of stem. Even these mild symptoms were completely obliterated three months after the initial challenge. Microscopic examination of thin section of the corm tissue revealed that in control plants fungal colonization was intense whereas significantly less infection was seen in transgenic plants (Figure 6). These observations clearly established that transgenic lines overexpressing *Petunia* defensins displayed high degree of resistance to Foc challenge.

### Copy Number and Expression Analysis of the Transgenes

To validate the stable integration of the transgenes in transformed tissues, selected transgenic lines were characterized by southern blotting. Genomic DNA was digested with restriction enzyme *Kpn*I as it cuts the T- DNA in the designed vector only once and hence the number of bands could be correlated with the copy number of the T-DNA in the transformed banana lines. T-DNA copy numbers ranging from 1 to 4 were seen in the different p*PhDef1*-1301 and p*PhDef2*-1301 transformed lines (Figure 7 A). To demonstrate genomic integrity of the two defensin expression cassettes, we performed two separate southern blots. When genomic DNAs of the transgenic banana plants were digested with *Eco*RI (which cuts inside the *Zea mays* polyubiquitin promoter and at the 3′ end of the nos terminator tagged with defensin genes) and probed with p*PhDef1* and p*PhDef2* coding sequence, expected hybridization signal of ∼1.2 kb were obtained in all the transgenic plants whereas the same was absent in the untransformed control plant. Further, when the genomic DNAs of same plants were digested using *Hin*dIII (which cuts at the 5′ end of the *Zea mays* polyubiquitin promoter) and *Kpn*I (which cuts at the 3′ end of the defensin genes) expected hybridization signal of ∼2.3 kb were obtained in all the transgenic plants whereas the same was absent in the untransformed control plant (Figure 7 B, C). Taken together, these results proved the genomic intactness of both the defensin expression cassettes in the transgenic plants analyzed.

Expression patterns of *PhDef1* and *PhDef2* genes in the transformed banana lines were analyzed by RT-PCR and Northern blotting. Total RNA was extracted from leaves and reverse transcribed to produce respective cDNAs. PCR amplification was carried out using primers specific to *PhDef1* and *PhDef2* to check the presence of *PhDef1* and *PhDef2* transcripts in the transgenic lines. A single expected product was amplified from the transgenic lines whereas no such product was amplified using cDNA derived from untransformed control plants (Figure 8 A, B). Northern blotting was performed by extracting total RNA from transgenic and untransformed control leaf tissue to confirm the transcription of *PhDef1* and *PhDef2* in the transgenic lines (Figure 8 C, D). Dense hybridization signals detected using DIG-labeled DNA probes generated using *PhDef1* and *PhDef2* coding sequences clearly indicated that the respective transgenes have stably integrated into the banana genome and expresses *PhDef1* and *PhDef2* mRNAs efficiently in transgenic banana plants.

## Discussion

In many developing countries around the world, banana is the staple food for millions of people where it serves as a rich source of carbohydrates, fiber, vitamins and minerals like phosphorus, calcium and potassium. Bananas are severely threatened by several debilitating diseases and pests which hinder the realization of full production potential for this economically important fruit crop. Also the constraints involved in banana breeding owing to the triploid nature of most elite cultivars limits the development of new cultivars with desirable traits [27].

The foremost challenge faced by the banana growing regions today is the development of pathogen resistant varieties which when available can reduce the loss resulting out of crippling disease pandemics especially those involving Fusarium wilt, black Sigatoka and banana bunchy top disease. The most prominent candidates for developing fungal resistance in crop plants as shown by a number of promising studies done over the past two decades are small antimicrobial peptides of plant origin collectively called defensins [28]. Several plant defensins have been found to be effective in controlling fungal diseases [29], [30], [31]. *NmDef02,* a leaf-derived defensin from tobacco, when expressed in potato, was found to be effective against *Phytophthora infestans* [32]. Similarly, radish seed defensin *Rs-AFP2* imparted enhanced resistance in rice against *Magnaporthe oryzae* and *Ralstonia solani* [33]. Alfalfa seed defensin *alfAFP* imparted resistance to potato plants against *Verticillium dahliae* [34]. Transgenic rice and melon overexpressing wasabi defensin (isolated from leaf tissues of *Wasabia japonica*) have been found to show durable resistance against *Magnaporthe grisea* [35] and *Alternaria solani*, *Fusarium oxysporum* [36] respectively. The fact that overexpression of such diverse defensins in different crop species resulted in durable resistance against damaging fungal pathogens clearly establishes the utility of defensins in developing resistance against economically important pathogens of food security crops like banana. Further, in the last few years novel defensins derived from soft floral tissues have been documented in great detail [37]. These floral defensins are unique in having 5 disulphide bonds and a C-terminal anionic prodomain [38]. Also, floral defensins derived from *Nicotiana alata* and *Petunia hybrida* have been demonstrated to show potent antifungal activity against *Fusarium oxysporum*. When the two *Petunia* floral defensins were overexpressed in transgenic banana plants using a strong constitutive promoter, high level resistance to Foc challenge was observed. Transgenic banana lines expressing either of the two defensins were significantly less chlorotic and appeared fresh weeks after the inoculation. The corm region of the banana plant which usually is the primary site of infection and colonization by Foc showed significantly less infestation and limited necrotic lesions. Further, in the corm region of the transgenic plants, occluding of xylem vessels as seen in a light microscope was negligible as compared to untransformed infected control plants. Both in vitro and ex vivo bioassays returned the same results wherein the untransformed control plants displayed characteristic wilting symptoms leading to their death within 6 weeks whereas the transgenic plants expressing the two *Petunia* defensins showed mild wilting symptoms initially but were able to recover fully later indicating superior resistance against *Fusarium* colonization.

The two *Petunia* defensins overexpressed in transgenic banana plants were tagged with *Zea mays* polyubiquitin promoter together with its intron containing 5′ UTR region to achieve the highest expression possible. *Zea mays* polyubiquitin promoter and 5′ UTR region have been documented in the past to give the highest expression levels in transgenic banana plants [39]. Southern blot analysis of the transgenic plants proved the stable integration of intact expression cassettes of both *PhDef1* and *PhDef2* in the banana genome. High level defensin transcript accumulation demonstrated by Northern blots of the transgenic plants signifies the importance of using efficient regulatory elements for generation of novel transgenic lines. Although the different transgenic lines analyzed had different copy numbers and different expression levels of the *Petunia* defensins, we failed to correlate these differences with the response of the plants to Foc infection. This could probably be attributed to their efficient anti-fungal activity at low concentrations as described before [18]. Embryogenic cells were used as the explants of choice for *Agrobacterium*-mediated genetic transformation as the transgenic plants derived from these cells are generally not expected to be chimeric which in turn guarantees stable expression of transgenes introduced into their genome [40]. Apart from their pure non-chimeric nature, another basic premise in the creation of transgenic plants with novel traits is generation of plants which are phenotypically similar to and show no growth altercation as compared to the untransformed controls. Transgenic banana plants expressing high level of the two *Petunia* defensins were phenotypically normal and no stunting was observed. High-level constitutive expression of these defensins did not induce any growth abnormality in the transgenic banana plants probably because the anionic C-terminal prodomains neutralize the cationic mature defensin domain which could otherwise lead to disturbances in the intracellular milieu ultimately leading to growth inhibition. Further, these C-terminal prodomains have also been predicted to contain vacuolar targeting signals. The targeting of these defensins to the vacuoles ensures proximity to their cognate proteases as well as it leads to compartmentalisation of these molecules which is essential, as they are often only needed when the cells are under attack from a pathogen.

Plant defensins, in general, work at the level of innate non-specific immunity against varied pathogens and are not related to the specific effector triggered immunity involving R-Avr protein interactions. Thus, the *Petunia* defensins characterized in this study can potentially be useful against race 4 of Foc as well as against other important phytopathogenic fungi infecting banana most importantly being black Sigatoka fungus *Mycosphaerella fijiensis*. Further, as genetic engineering involves the introduction of desirable characters like resistance to most damaging plant pathogens in proven elite cultivars, the genes assembled for imparting resistance against diverse pathogens could also be inserted into a single binary vector so that the transgenics resulting from this kind of gene stacking are capable of tolerating multiple pathogens at the same time which in fact is more of a rule than an exception in a farmer’s field [41]. Development of efficient resistance against Foc in banana plants using the *Petunia* floral defensins can potentially be utilized in gene stacking for obtaining sustainable resistance towards major pathogens of this important food crop.
